# Foxp3^+^ Regulatory T Cells in Mouse Models of Type 1 Diabetes

**DOI:** 10.1155/2013/940710

**Published:** 2013-03-14

**Authors:** Cathleen Petzold, Julia Riewaldt, Deepika Watts, Tim Sparwasser, Sonja Schallenberg, Karsten Kretschmer

**Affiliations:** ^1^Center for Regenerative Therapies Dresden, 01307 Dresden, Germany; ^2^Institute of Infection Immunology, TWINCORE/Centre for Experimental and Clinical Infection Research, 30625 Hanover, Germany; ^3^Paul Langerhans Institute Dresden, German Center for Diabetes Research (DZD), 01307 Dresden, Germany

## Abstract

Studies on human type 1 diabetes (T1D) are facilitated by the availability of animal models such as nonobese diabetic (NOD) mice that spontaneously develop autoimmune diabetes, as well as a variety of genetically engineered mouse models with reduced genetic and pathogenic complexity, as compared to the spontaneous NOD model. In recent years, increasing evidence has implicated CD4^+^CD25^+^ regulatory T (Treg) cells expressing the transcription factor Foxp3 in both the breakdown of self-tolerance and the restoration of immune homeostasis in T1D. In this paper, we provide an overview of currently available mouse models to study the role of Foxp3^+^ Treg cells in the control of destructive *β* cell autoimmunity, including a novel NOD model that allows specific and temporally controlled deletion of Foxp3^+^ Treg cells.

## 1. Introduction

Type 1 diabetes (T1D) is a chronic disease manifested by the loss of functional insulin producing *β* cells of pancreatic islets, caused by islet infiltrating self-reactive CD4^+^ and CD8^+^ T cells that mediate *β*-cell destruction [1]. Many of the immunological aspects of human T1D are mimicked by the nonobese diabetic (NOD) mouse model, which shows islet infiltration and destructive autoimmune insulitis as early as four weeks of age and spontaneously progresses to overt diabetes in the adult [2]. Observations in mice and humans have demonstrated that CD4^+^CD25^+^ regulatory T (Treg) cells expressing the forkhead box transcription factor Foxp3 play an indispensable role in the maintenance of immune homeostasis by regulating inflammatory responses against invading pathogens and preventing destructive autoimmunity [3–6]. A particularly striking example of Foxp3^+^ Treg cell function that restrains destructive tissue-specific autoimmune responses is the observation that acute ablation of Treg cells in adult NOD mice carrying a pancreatic *β* cell-reactive T cell receptor (TCR) as a transgene unleashes overt autoimmune diabetes within days (see Section 4.3). Given their nonredundant function in maintaining immune homeostasis, it is not surprising that Foxp3^+^ Treg cells have attracted considerable attention as particularly promising gain-of-function targets in clinical settings of unwanted immune responses, such as T1D. Here, we provide an overview of mouse models for T1D that, in our view, appear particularly suitable to study various aspects of Foxp3^+^ Treg cell-mediated control of *β* cell autoimmunity, ranging from classical diabetes models adapted to the functional analysis of Treg cells to novel genetic tools for Treg cell depletion in NOD mice.

## 2. Pancreatic **β** Cell Expression of Neo-Self-Antigens

### 2.1. Spontaneous Models

Double-transgenic mice that coexpress model antigens (such as ovalbumin, LCMV glycoprotein, or influenza hemagglutinin; HA) in pancreatic *β* cells together with TCRs reactive to the respective *β* cell neo-self-antigen (either MHC class I- or class II-restricted) spontaneously develop autoimmune diabetes, recapitulating some aspects of the spontaneous NOD model, albeit with faster kinetics [8]. As an example, transgenic expression of an HA-reactive TCR on CD4^+^ (TCR-HA_107–119_) [9, 10] or CD8^+^ (CL4-HA_512–520_) [11] T cells promotes spontaneous diabetes development in mice that additionally express HA under control of the rat insulin promoter (RIP-HA) [12]. Potential limitations of the RIP-HA model, many of which are shared between the various double-transgenic diabetes models, have been discussed in detail elsewhere [13]. Nevertheless, TCR-HA × RIP-HA mice offer some advantages that appear particularly relevant in the context of mechanistic studies on antigen-specific tolerance induction. While limiting *β* cell pathogenicity to a single, well-defined neo-self-protein, and in contrast to many other transgenic TCRs (e.g., DO11.10), the TCR-HA is expressed only on a fraction of CD4^+^ T cells (ranging from 5% to 20% in different lymphoid tissues) that coexist with polyclonal populations of TCR-HA^−^ CD4^+^ T cells expressing endogenous TCR gene rearrangements [14]. In the TCR-HA × RIP-HA model, selective delivery of agonist ligand to steady-state DEC-205^+^ DCs has been shown to interfere with the development of autoimmune diabetes [15], probably due to the extrathymic induction of antigen-specific Foxp3^+^ Treg cells from initially naïve Foxp3^−^TCR-HA^+^ T cells [16, 17]. However, it appears desirable that findings observed in double-transgenic models of spontaneous autoimmune diabetes will subsequently be extended to the nontransgenic NOD model.

### 2.2. Adoptive Transfer Models

**Figure 1:**
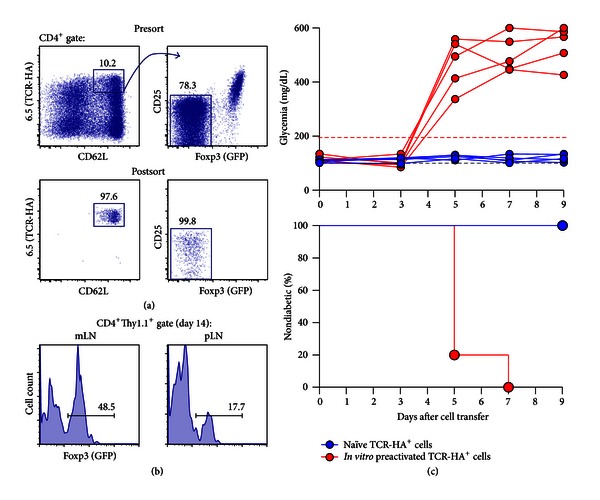
Adoptive TCR-HA^+^ T cell transfer into immunocompetent RIP-HA mice. (a) Using the clonotypic antibody 6.5, naïve TCR-HA^+^ T cells (CD4^+^6.5^+^CD62L^high^ CD25^−^GFP^−^) were FACS purified from BALB/c.Thy1.1 TCR-HA × Foxp3^IRES-GFP^ mice, after CD4 bead enrichment of pooled cells from spleen and LNs. Presort (top) and postsort (bottom) analyses of TCR-HA/CD62L (left) and CD25/GFP (right) expression among CD4-gated cells are depicted. The gating scheme is illustrated by the line with arrowhead. For antigen-specific stimulation *in vitro*, TCR-HA^+^ T cells were cultured as previously described [7], in the presence of HA_107–119_ peptide (10 *μ*g/mL). As indicated, naïve or *in vitro* preactivated TCR-HA^+^ T cells were injected i.v. into immunocompetent BALB/c.Thy1.2 RIP-HA mice. (b) Flow cytometry of Foxp3^IRES-GFP^ expression among gated CD4^+^Thy1.1^+^ cells at day 14 after adoptive transfer into BALB/c.RIP-HA recipient mice (mLN: mesenteric lymph node; pLN: pancreatic LN). Numbers in dot plots or histograms indicate the percentage of cells in the respective gate. (c) Blood glucose concentrations (top) and diabetes incidence (bottom) of BALB/c.RIP-HA mice injected with naïve (blue circles, *n* = 5) or *in vitro* preactivated TCR-HA^+^ T cells (red circles, *n* = 5). Blood glucose concentrations (in mg/dL) of individual mice were determined every other day and plotted against time. The grey dashed line indicates normoglycemia. Mice were considered diabetic at blood glucose levels above 200 mg/dL (red dashed line) on at least two consecutive measurements or with blood glucose levels once above 400 mg/dL.

In immunodeficient (Rag^−/−^, *nude*) RIP-HA recipient mice, adoptive transfer of naïve CD4^+^TCR-HA^+^ T cells (i.e., without prior T cell activation *in vitro*) from TCR-HA donor mice induces autoimmune diabetes within 1-2 weeks [10, 14, 18]. In immunocompetent recipient mice, naïve CD4^+^TCR-HA^+^ T cell transfer (Figure 1(a)) fails to induce overt diabetes (Figure 1(c)), perhaps due to extrathymic induction of a Foxp3^+^ Treg cell phenotype in a significant proportion of initially Foxp3^−^ T cells, upon recognition of the cognate antigen on antigen-presenting cells residing in peripheral lymphoid tissues (Figure 1(b)). Notably, initially naïve CD8^+^CL4^+^ [19] and CD4^+^TCR-HA^+^ (Figure 1(c)) T cells, which had been preactivated *in vitro* as previously described [7], can promote autoimmune diabetes development shortly after injection into immunocompetent recipient mice. It is important to emphasize that kinetics and efficiency of diabetes induction critically depend on suitable culture conditions for preactivation.

Double-transgenic TCR-HA × Pgk-HA mice represent a convenient source of antigen-specific Foxp3^+^ Treg cells, as expression of HA under control of the phosphoglycerate kinase promoter (Pgk-HA) results in peripheral accumulation of intrathymically induced Foxp3^+^TCR-HA^+^ Treg cells [22]. Foxp3^−^TCR-HA^+^ T regulatory 1 cells with potent suppressor capacity can be readily isolated from peripheral lymphoid tissues of TCR-HA mice that coexpress HA under the control of the Ig-*κ* promoter [23]. Overall, the RIP-HA model offers unique opportunities to study mechanisms of antigen-specific suppression of *β* cell autoimmunity, employing cotransfer of TCR-HA^+^ Treg cells, either with a Foxp3^+^ or Foxp3^−^ phenotype, together with pathogenic T effector cells (CD4^+^TCR-HA^+^ or CD8^+^CL4^+^).

## 3. NOD Adoptive Transfer Models

### 3.1. Adoptive BDC2.5 T Cell Transfer

**Figure 2:**
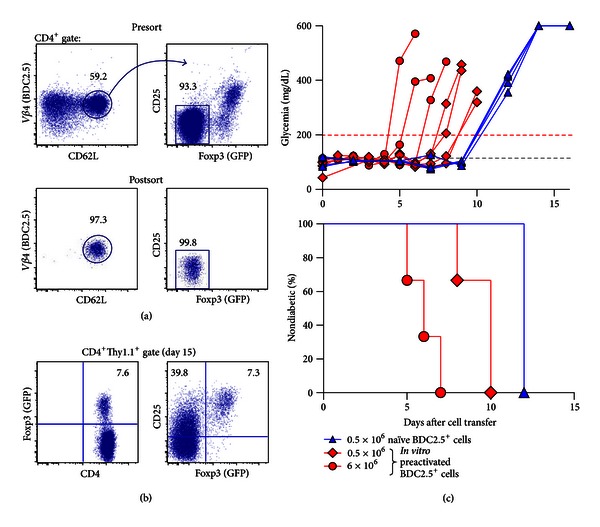
Adoptive BDC2.5^+^ T cell transfer into immunodeficient NOD mice. (a) Using anti-V*β*4 antibodies, naïve BDC2.5^+^ T cells (CD4^+^V*β*4^+^CD62L^high^ CD25^−^GFP^−^) were FACS purified from NOD.Foxp3^DTR-GFP^  ×BDC2.5 mice, after CD4 bead enrichment of pooled cells from spleen and LNs. Presort (top) and postsort (bottom) analyses of V*β*4/CD62L (left) and CD25/GFP (right) expression among CD4-gated cells are depicted. The gating scheme is illustrated by the line with arrowhead. For antigen-specific stimulation *in vitro*, BDC2.5^+^ T cells were cultured as previously described [7], in the presence of the mimotope peptide RTRPLWVRME (10 *μ*g/mL). Naïve or *in vitro* preactivated BDC2.5^+^ T cells were injected i.v. into NOD.Rag1^−/−^ recipient mice, as indicated below. (b) Flow cytometry of GFP (left) and CD25/GFP (right) expression among gated CD4^+^ cells from LNs at day 15 after adoptive transfer into NOD.Rag1^−/−^ recipient mice. Numbers in dot plots indicate the percentage of cells in the respective quadrant or gate. (c) Blood glucose concentrations (top) and diabetes incidence (bottom) of NOD.Rag1^−/−^ recipient mice injected with naïve (5 × 10^5^ cells/mouse, blue triangles, *n* = 4) or *in vitro* preactivated BDC2.5^+^ T cells (5 × 10^5^ cells/mouse, red squares, *n* = 4; or 6×10^6^ cells/mouse, red circles, *n* = 3). Blood glucose concentrations of recipient mice were determined and plotted as described in the legend for Figure 1.

CD4^+^ T cells expressing the BDC2.5 TCR as a transgene, which is reactive to islet *β* cells in the context of MHC class II Ag7 molecules, are highly diabetogenic in NOD mice [24, 25]. While agonistic mimotope peptides that stimulate BDC2.5^+^ T cells at nanomolar concentrations had been described some years ago [26], chromogranin A has only recently been proposed to represent the natural self-antigen responsible for pancreatic *β* cell pathogenicity of BDC2.5^+^ T cells [27]. Naïve BDC2.5^+^ T cells, FACS purified (Figure 2(a)) from peripheral lymphoid tissues of immunocompetent NOD.BDC2.5 mice with Foxp3-dependent GFP expression (see Section 4.3) and adoptively transferred into either TCR-*β* ^−/−^ [28] or Rag1^−/−^ (Figure 2(b)) NOD mice, undergo lymphopenia-driven proliferation, resulting in the acquisition of a Foxp3^+^ Treg cell phenotype in a significant proportion of initially Foxp3^−^ T cells [29–31]. Nevertheless, without prior T cell activation *in vitro*, adoptive transfer of 5 × 10^5^ naïve BDC2.5^+^ T cells consistently induces autoimmune diabetes in lymphopenic NOD mice within 13.0 ± 1.2 days, as revealed by high blood glucose concentrations (Figure 2(c)). In this adoptive transfer model, autoimmune diabetes onset can be further accelerated by TCR prestimulation *in vitro* and injection of increasing numbers of BDC2.5^+^ T cells (Figure 2(c)). In fact, adoptive transfer of *in vitro* activated BDC2.5^+^ T cells into neonatal or immunodeficient (scid, TCR-*β* ^−/−^, Rag1^−/−^) NOD recipient mice is commonly used as a standard protocol for the induction of autoimmune-mediated pancreatic *β* islet inflammation. In contrast to T helper (Th) 2 [32] and Th17 [33] cells that had been generated from BDC2.5^+^ T cells *in vitro*, Th1-polarized BDC2.5^+^ T cells efficiently induce aggressive autoimmune diabetes upon injection into neonatal NOD mice [32], whereas Th17 BDC2.5^+^ cells have been reported to promote rapid onset of diabetes in adult NOD.scid mice [33].

**Figure 3:**
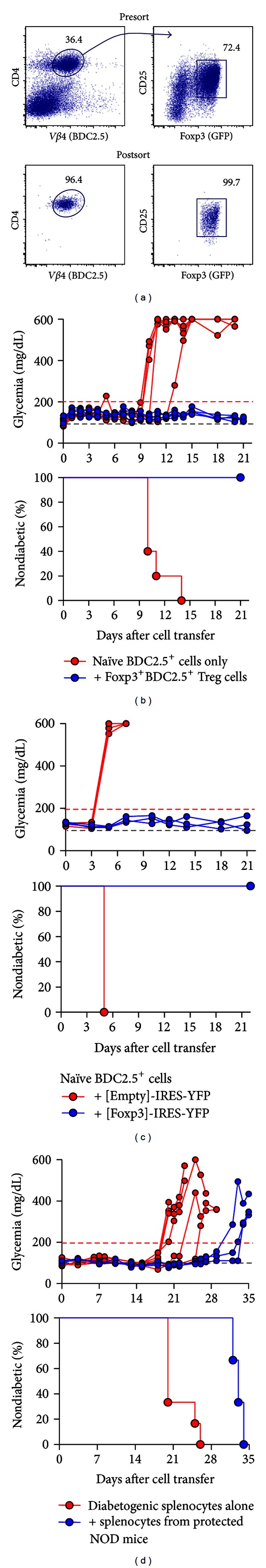
Foxp3^+^ Treg cells in NOD transfer models. (a–c) Adoptive BDC2.5^+^ T cell transfer. (a) FACS purification of BDC2.5^+^Foxp3^+^ Treg cells (CD4^+^V*β*4^+^CD25^+^GFP^+^) from pooled spleen and LNs of NOD.Foxp3^Cre-GFP^  × BDC2.5 mice after magnetic bead enrichment of CD25^+^ cells. Presort (top) and postsort (bottom) analyses of CD4/V*β*4 (left) and CD25/GFP (right) expression among gated lymphocytes are depicted. The gating scheme is illustrated by the line with arrowhead. Numbers in dot plots indicate the percentage of cells in the respective gate. (b) For diabetes induction, NOD.Rag1^−/−^ recipient mice were injected with naïve BDC2.5^+^ T cells (5 × 10^5^ cells/mouse), either alone (red circles, *n* = 5) or coinjected with Foxp3^+^BDC2.5^+^ Treg cells (5 × 10^4^ cells/mouse, blue circles, *n* = 5) that had been FACS purified as shown in (a). See Figure 2(a) for details on the flow cytometric isolation of naïve BDC2.5^+^ T cells. (c) In addition to naïve BDC2.5^+^ T cells (5 × 10^5^ cells/mouse), NOD.Rag1^−/−^ recipient mice were coinjected with 1 × 10^5^ BDC2.5^+^ T cells that exhibited retrovirus-mediated expression of either [Empty]-IRES-YFP (red circles, *n* = 3) or [Foxp3]-IRES-YFP (blue circles, *n* = 3). Retrovirus infections of initially naïve, TCR stimulated BDC2.5^+^ T cells were performed essentially as described previously [20]. (d) Adoptive transfer of polyclonal T cells. NOD.Rag1^−/−^ recipient mice received splenocytes harvested from diabetic NOD donor mice (red circles, *n* = 6, average diabetes development at day 21.8 ± 2.6) or were coinjected with equivalent numbers of splenocytes from NOD donors that maintained normoglycemia until 26 weeks of age after treatment with recombinant anti-DEC-205 antibodies fused to whole proinsulin, beginning at 7 weeks of age (blue circles, *n* = 3, average diabetes development at day 33.0 ± 0.8) (adopted from [21]). Blood glucose concentrations of recipient mice in (b–d) were determined and plotted as described in the legend for Figure 1.

In addition to providing diabetogenic CD4^+^BDC2.5^+^ T effector cells, NOD.BDC2.5 mice with Foxp3-dependent GFP expression [28, 34–37] represent a convenient source of Foxp3^+^ Treg cells with the same antigen specificity, which can be readily FACS purified (V*β*4^+^CD4^+^CD25^+^GFP^+^) from CD25 bead enriched single cell suspensions of peripheral lymphoid donor tissues (Figure 3(a)). Importantly, cotransfer of as few as 5 × 10^4^ Foxp3^+^BDC2.5^+^ Treg cells is sufficient to mediate long-term autoimmune protection of NOD.Rag1^−/−^ mice that additionally received 5 × 10^5^ diabetogenic naïve BDC2.5^+^ T cells (Figure 3(b)). Besides studies on the suppressor function of Foxp3^+^BDC2.5^+^ Treg cell populations naturally developing in NOD.BDC2.5 mice, the adoptive BDC2.5^+^ T cell transfer model provides the opportunity to assess the suppressive capacity of Foxp3^+^ Treg cells that had been artificially generated from initially Foxp3^−^BDC2.5^+^ T cells in experimental settings of extrathymic Treg cell induction, for example, by retrovirus-mediated ectopic expression of Foxp3 (Figure 3(c)). Note that, as compared to the adoptive transfer of naïve BDC2.5^+^ T cells alone (Figure 3(b)), cotransfer of [Empty]-IRES-YFP^+^ BDC2.5^+^ T cells substantially accelerates diabetes due to T cell prestimulation *in vitro* for retrovirus infection (Figure 3(c)).

In immunocompetent NOD mice, the *in vivo *application of *in vitro *expanded Foxp3^+^BDC2.5^+^ Treg cells [38, 39], as well as Foxp3^+^BDC2.5^+^ Treg cells, generated *in vitro* either by ectopic expression of Foxp3 [20] or TGF-*β*-mediated induction of Foxp3 expression [40], can be effective in prevention or even reversal of spontaneously developing diabetes.

### 3.2. Adoptive Transfer of Polyclonal T Cells

Unfractionated splenocytes from diabetic, non-TCR transgenic NOD donor mice can induce autoimmune diabetes within 3 weeks after injection into immunodeficient NOD mice, such as NOD.Rag1^−/−^ mice (Figure 3(d)) or irradiated NOD mice [41]. Although the relative contribution of CD4^+^ and CD8^+^ T cells had remained controversial in previous studies [42, 43], more recent observations in NOD.scid mice using highly purified T cell populations revealed that the development of autoimmune diabetes in this adoptive transfer model requires both CD4^+^ and CD8^+^ T cells [44]. Cotransfer of polyclonal Foxp3^+^ Treg cells, either purified populations or contained in unfractionated total cell populations, can be employed to assess their suppressive capacity in the context of autoimmune diabetes. After tolerogenic DEC-205^+^ dendritic cell vaccination to promote proinsulin-reactive Foxp3^+^ Treg cell activity, cotransfer of total spleen cells from autoimmune protected NOD donors can delay the onset of diabetogenic splenocyte-mediated diabetes in NOD.Rag1^−/−^ recipients (Figure 3(d)) [21].

## 4. Abrogation of Foxp3^+^ Treg Cell Activity

### 4.1. Genetic Deficiency

Abrogated Treg cell function has been actively debated as a putative mechanism underlying various autoimmune disorders in humans [45]. The important role of Foxp3^+^ Treg cells in protection from autoimmune diabetes is highlighted by the notion that T1D represents a major component of the IPEX (immune dysfunction, polyendocrinopathy, enteropathy, X-linked) syndrome [46–48] that affects humans with abrogated Treg cell function due to mutations in the FOXP3 gene [49–51]. In mice, spontaneous [52] or gene-targeted [53] Foxp3 deficiency leads to death by 3-4 weeks of age due to the development of a fatal multiorgan autoimmune syndrome that recapitulates many clinical features of the human IPEX syndrome. Notably, the manifestation of autoimmune diabetes in Foxp3-deficient mice on non-autoimmune-prone genetic backgrounds has not been reported thus far. Moreover, Foxp3-deficient mice on the diabetes-prone NOD background develop exocrine pancreatitis and peri-insulitis, but do not manifest invasive insulitis and diabetes [54]. Several nonmutually exclusive mechanisms may account for the absence of overt diabetes in Foxp3-deficient mice, which includes premature death and altered T cell repertoire selection due to severe defects in thymic T cell development [55]. In any case, this striking difference to human IPEX patients regarding the manifestation of autoimmune diabetes limits the exploitation of mice with constitutive genetic Foxp3 deficiency and concomitant absence of functional Treg cells in studies on pancreatic *β* cell autoimmunity.

### 4.2. Administration of Anti-CD25 mAbs

To examine the contribution of Foxp3^+^ Treg cells in the control of pancreatic *β* cell autoimmunity, administration of anti-CD25 mAbs has been widely used as a loss-of-function approach, with the overwhelming majority of studies employing the clone PC61 (rather than 7D4). Whether abrogation of suppressor activity upon *in vivo* administration of anti-CD25 mAbs can be attributed to the functional inactivation [56] or the actual physical elimination (deletion) of CD25-expressing Foxp3^+^ Treg cells has been controversially discussed [56–58]. In otherwise nonmanipulated NOD mice, single dose [59] or repeated [60] injection of the anti-CD25 mAb PC61 can significantly accelerate the spontaneous development of autoimmune diabetes in adolescent but not adult [61] females. In experimental settings of tolerogenic regimens that result in long-term protection of NOD mice from autoimmune *β* cell destruction, anti-CD25 mAbs have been employed as an approach to address the relative contribution of CD25^+^ Treg cells in tolerance induction, with PC61 administration resulting either in the rapid precipitation of overt diabetes [59, 61–63] or the failure to break established *β* cell tolerance and maintenance of normoglycemia [64–66]. However, interpretation of results from such experiments is hampered by the fact that CD25 expression is not exclusive to Foxp3^+^ Treg cells. In fact, PC61 administration to adult NOD mice has also been reported to delay diabetes onset [65], perhaps due to its negative impact on activated CD4^+^ and CD8^+^ T effector cells with upregulated CD25 expression. Additionally, it appears important to emphasize that anti-CD25 treatment with the aim to interfere with Treg cell function, either by deletion or functional inactivation, will inevitably spare Foxp3^+^ Treg cells with a CD25^low/−^ phenotype. Consistently, anti-CD25 treatment protocols preserve significant numbers of Foxp3^+^ cells [56–58, 67, 68].

### 4.3. Diphtheria Toxin-Mediated Deletion of Foxp3^+^ Treg Cells

**Figure 4:**
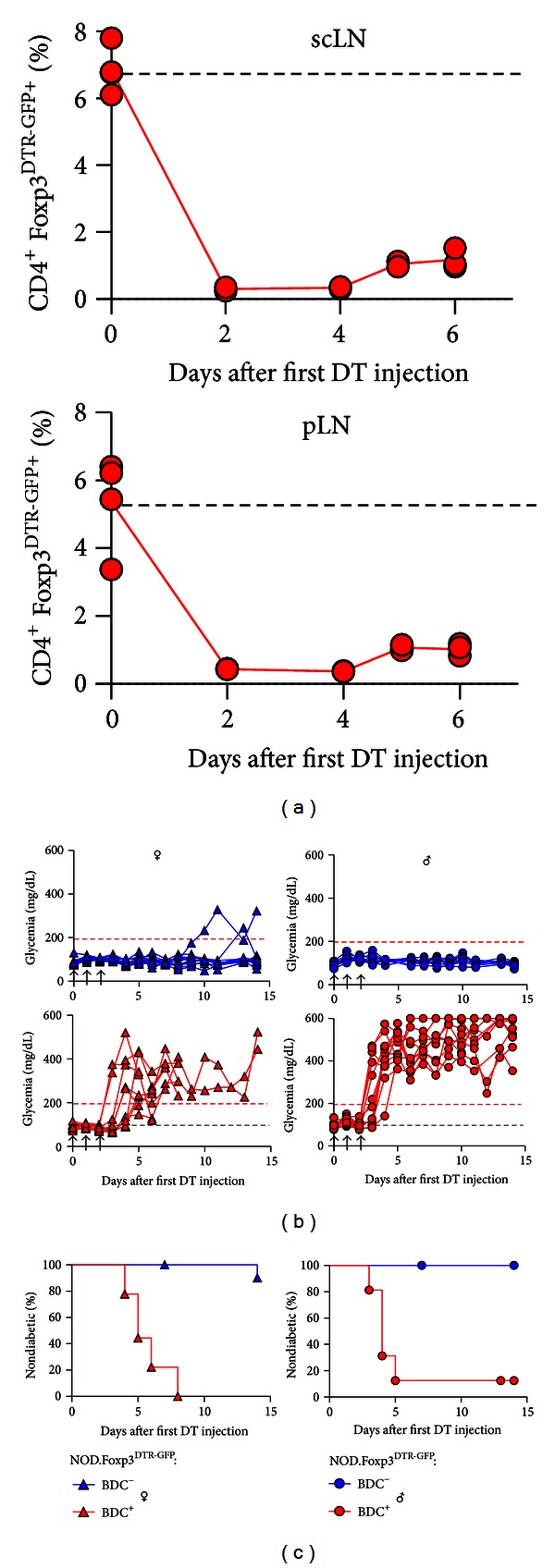
Foxp3^+^ Treg cell ablation in NOD.Foxp3^DTR-GFP^ mice. (a) Percentage of Foxp3^DTR-GFP+^ cells among CD4^+^ T cells in subcutaneous lymph nodes (scLN, top) and pancreatic LNs (pLN, bottom) of NOD.Foxp3^DTR-GFP^  × BDC2.5 mice, which were either left untreated (dashed line) or i.p. injected with DT (0.5 *μ*g/mouse on 3 consecutive days). Symbols represent individual mice at indicated time points after the first DT administration. (b) Blood glucose concentrations and (c) diabetes incidence of NOD.Foxp3^DTR-GFP^ mice (blue triangles: females, *n* = 11; blue circles: males, *n* = 11) and NOD.Foxp3^DTR-GFP^  ×BDC2.5 mice (red triangles: females, *n* = 9; red circles: males, *n* = 12), after DT administration, as indicated by the arrowheads.

Foxp3-dependent expression of the human diphtheria toxin (DT) receptor as a transgene, either from an internal ribosome entry site (IRES) downstream of the Foxp3coding region [69] or from a Foxp3 bacterial artificial chromosome (BAC) (termed “depletion of regulatory T cell” mice, DEREG; [36]), provides an opportunity for specific and temporally controlled deletion of Foxp3^+^ Treg cells in mice on non-autoimmune-prone genetic backgrounds. In both mouse models, Foxp3^+^ Treg cell depletion by the *in vivo* administration of DT promotes the development of autoimmune disorders, albeit with differences in the severity of autoimmune symptoms [36, 69]. On the NOD genetic background, two independent mouse lines with DT receptor expression selectively in Foxp3^+^ Treg cells have been generated. While Feuerer et al. established a novel Foxp3 BAC transgenic line employing NOD embryos [28], we generated NOD.Foxp3^DTR-GFP^ mice by backcrossing the BAC-Foxp3^DTR-GFP^ transgene of the well-characterized DEREG mouse model [36, 70–73] onto the NOD/Lt background (Figure 4).

Transgenic expression of the BDC2.5 TCR efficiently prevents the development of spontaneous autoimmune diabetes in immunocompetent NOD females [74] but dramatically accelerates diabetes progression in immunodeficient NOD mice, such as NOD.TCR-*β* ^−/−^ or NOD.Rag1^−/−^ mice [74], as well as in NOD.Foxp3^−/−^ mice [54]. Acute ablation of Foxp3^+^ Treg cells (Figure 4(a)) can lead to transiently increased blood glucose concentration in some adult NOD.Foxp3^DTR-GFP^ females, but fails to consistently promote overt diabetes (Figure 4(b)). In NOD.Foxp3^DTR-GFP^  × BDC2.5 females, Foxp3^+^ Treg cell ablation triggers autoimmune *β* cell destruction within 8 days after initiation of DT administration (Figure 4(b)). Notably, and in contrast to the spontaneous NOD model, the NOD.Foxp3^DTR-GFP^  × BDC2.5 model additionally allows the induction of autoimmune diabetes in male mice, with similar efficiency and kinetics as compared to females (Figure 4(c)).
